# Acoustic and optoacoustic stimulations in auditory brainstem response test in salicylate induced tinnitus

**DOI:** 10.1038/s41598-023-39033-5

**Published:** 2023-07-24

**Authors:** Katayoon Montazeri, Mohammad Farhadi, Zeinab Akbarnejad, Abdoreza Asadpour, Abbas Majdabadi, Reza Fekrazad, Saeid Mahmoudian

**Affiliations:** 1 ENT and Head and Neck Research Center, The Five Senses Health Institute, School of Medicine, Hazrate Rasoul Akram Hospital, Iran University of Medical Sciences, Tehran, 1445613131 Iran; 2 Intelligent Systems Research Centre, Ulster University, Derry Campus, Derry~Londonderry, Northern Ireland UK; 3 Laser Research Center of Dentistry, Dentistry Research Institute, Tehran University of Medical Sciences, Tehran, Iran; 4 Radiation Sciences Research Center, Laser Research Center in Medical Sciences, AJA University of Medical Sciences, Tehran, Iran

**Keywords:** Neuroscience, Medical research, Neurology

## Abstract

As a common debilitating disorder worldwide, tinnitus requires objective assessment. In the auditory brainstem response (ABR) test, auditory potentials can be evoked by acoustic or optoacoustic (induced by laser light) stimulations. In order to use the ABR test in the objective assessment of tinnitus, in this study, acoustic ABR (aABR) and optoacoustic ABR (oABR) were compared in the control and tinnitus groups to determine the changes caused by sodium salicylate (SS)-induced tinnitus in rat. In both aABR and oABR, wave II was the most prominent waveform, and the amplitude of wave II evoked by oABR was significantly higher than that of aABR. Brainstem transmission time (BTT), which represents the time required for a neural stimulation to progress from the auditory nerve ending to the inferior colliculus, was significantly shorter in oABR. In the tinnitus group, there was a significant increase in the threshold of both ABRs and a significant decrease in the amplitude of wave II only in the oABR. Based on our findings, the ABR test has the potential to be used in the assessment of SS-induced tinnitus, but oABR has the advantages of producing more prominent waveforms and significantly reducing the amplitude of wave II in tinnitus.

## Introduction

Tinnitus or the phantom perception of sound, is a common and annoying symptom which its neural basis is still unclear. The prevalence of tinnitus is the same in both sexes but increases with age^1^. It is estimated that 14% of adults experience some form of tinnitus in their lifetime, 2% of which is severe. It has been confirmed that in tinnitus, secondary compensatory changes occur in the central nervous system as a result of the primary defect in the peripheral auditory system^2,3^. An increase in neural firing rate, plastic changes in the tonotopic organization of the brain cortex, and an increase in neural synchrony are among the central changes in tinnitus^4^. Unfortunately, despite the various medications and methods used to treat tinnitus, no cure has been accepted as the gold standard. In addition, there is a lack of objective tools in the evaluation of tinnitus, and in most cases, the tools for evaluating tinnitus include visual analog scales, audiometric tests, questionnaires, and psychometric tinnitus tests, which are all subjective tools and rely on self-report^5,6^. A systematic review of objective measures of tinnitus reported that the overall quality of the evidence was low and that there were no reliable or reproducible objective measures of tinnitus^7^. Therefore, there is a need for an objective tool that is not based on patients’ statement and is less influenced by interfering factors. Auditory brainstem response (ABR) is a non-invasive test that records the auditory evoked potentials via surface electrodes which placed on the skull. In the ABR test, five to six consecutive positive peak waves are recorded with an interval of about 0.8 ms, which shows the neural activity of the auditory pathway from the cochlea and auditory nerve to the brainstem. The ABR test is sensitive to factors affecting conduction velocity and waveform delay. It has also been shown that in neural desynchronization, the amplitude of ABR peaks decreases or disappears^8^. In the last decade, efforts have been made to determine the changes caused by tinnitus in the ABR test in the hope that this test can be used in objective evaluation of tinnitus. The results of these efforts were summarized in two systematic reviews, one of which performed in humans and the other in rat animal model and both reported a high level of heterogeneity among studies that used ABR test in the assessment of tinnitus. This discrepancy may be due to the use of different protocols for electrophysiological recording, and/or because tinnitus-induced changes in the ABR were not sufficiently significant^9,10^. It has been confirmed in human and animal species, visible and infrared (IR) lights have the ability to penetrate through the skull, and in the auditory system, in addition to the therapeutic and regenerative effects, they also have the ability to stimulate target neuron groups with high selectivity^11–15^. The elicitation of auditory evoked potentials by light photons is called “intracochlear light stimulation” or “optoacoustic stimulation” which was initially proposed as a possible alternative to electrical stimulation in cochlear implants (CI)^16^. To improve the performance of CI, light which has a high spatial resolution, has no artifact and do not require direct contact with tissue has been proposed as an alternative to electric current^17,18^. As pioneers, Izzo et al. compared compound action potentials (CAPs) evoked by acoustic and optoacoustic stimulations^19,20^. Since then, several studies have used laser light to evoke auditory potentials and the results were recorded as CAP or ABR^16,18,21–24^. Two main mechanisms proposed for optoacoustic stimulation include the photothermal, and the optoacoustic effects. In the photothermal effect, the absorption of light activates heat-sensitive ion channels and evokes a neural response. In the optoacoustic effect, absorption of light by cellular photoacceptors leads to a change in temperature and an increase in volume, followed by shrinkage, which produces a pressure wave that mechanically stimulates cochlear hair cells^25^. The hair cells of cochlea must be intact to transmit this pressure wave, so optoacoustic effect will be impossible in deaf animals. Currently, although optoacoustic stimulation has been performed in deaf animals in some cases^26^, most studies have reported the optoacoustic effect as the main mechanism of auditory evoked potentials^16–23,27^. The studies that have investigated the parameters of acoustic and optoacoustic stimulations in ABR, have reported an increase in wave amplitude with increasing sound intensity or pulse energy, and have also confirmed reaching a saturation level with increasing pulse energy in oABR^26,28^. In optoacoustic studies, ear surgery has been performed to access the cochlea and stimulate hair cells, which may be due to the narrow and angled external auditory canal in animal models^16–20^. However, it has been shown that to record laser-evoked auditory potentials in humans, an optical fiber can be guided through the external auditory canal, so future oABR recordings in humans will not require surgery^29^. The ability of light to evoke auditory potentials can open a new horizon for understanding the essential neural correlates involved in tinnitus. Given the heterogeneity in acoustic ABR (aABR) findings in tinnitus, we hypothesized that optical stimulation may enhance characteristics of auditory evoked potentials due to the high spatial selectivity and velocity of light which may results in sharper ABR waveforms and shorter brainstem transmission time (BTT).

In the current study, IR pulsed laser was used for cochlear optoacoustic stimulation (“optophony”) in sodium salicylate (SS)-induced tinnitus rats. It has been reported that intraperitoneal injection of SS with a certain dose can cause tinnitus in the rat animal mode^30^. It has been found that the induction of tinnitus by SS at a given dose can be quite reliable, while the induction of tinnitus by noise may have variable success^31^. Therefore, we chose SS injection as the tinnitus induction method for the first experiment. SS affects not only the cochlea and auditory nerve, but also some central auditory circuits (inferior colliculus, auditory cortex) and may cause tinnitus and hearing loss. SS passes the blood–brain barrier and causes an imbalance between the excitatory and inhibitory circuits, which leads to changes in the auditory response characteristics of neurons. SS injection has been reported to significantly reduce lateral wall stiffness of outer hair cells (OHCs) and the degree of hearing loss depends on the extent and severity of damage to the OHCs^32^. It has been confirmed that the hyperactivity associated with tinnitus is primarily related to the auditory cortex and then to extraleminscal structures and the brainstem. In most cases, tinnitus is associated with hearing loss, but in some cases, tinnitus is associated with “hidden hearing loss” caused by cochlear synaptopathy. Clinically, these people have normal hearing, but they have problems in noisy environments^33^. It should be noted that only the ABR changes caused by SS were investigated here and changes caused by other tinnitus induction methods should be investigated in future studies. According to the ethical rules, the minimum acceptable number of animals were placed in the experimental groups, which is one of the limitations of this research.

Here, aABR and oABR tests were performed in control and SS-induced tinnitus groups of rat animal model. The purpose of the study was to compare the waveform characteristics in the control and tinnitus groups to determine whether oABR has an advantage over aABR due to the high spatial selectivity and high velocity of light.

## Results

### Behavioral test

The behavioral test of gap pre-pulse inhibition of acoustic startle (GPIAS) was performed for both groups, which includes the gap-in-noise (GIN) ratio to indicate the occurrence of tinnitus and the Pre-pulse Inhibition (PPI) ratio to assess the hearing status of the animal^34^.

### Between-groups and within-group comparisons

The mean ± standard deviation (M ± SD) of the GIN ratio in the tinnitus group after the SS injection period was 20.83 ± 8, which was significantly lower than 44.05 ± 10 in the control group (P = 0.002). The M ± SD of the GIN ratio in the tinnitus group before tinnitus induction was 41.61 ± 8.3, after which it significantly decreased to 20.83 ± 8 (P = 0.01). PPI ratio did not show any significant difference either between tinnitus and control groups or before and after tinnitus induction in tinnitus group (Table 1).

**Table 1:** Between-groups and within-group comparisons of behavioral test (GPIAS).

GPIAS test			
	Control group (M ± SD)	Tinnitus group (after SS) (M ± SD)	*P* value
Between-groups (t-test)			
GIN ratio (%)	44.05 ± 10	20.83 ± 8	**0.002**
PPI ratio (%)	95.68 ± 1.9	96.54 ± 2.2	0.78

	Tinnitus group (before SS)	Tinnitus group (after SS)	
Within-group (paired-t-test)			
GIN ratio (%)	41.61 ± 8.3	20.83 ± 8	**0.01**
PPI ratio (%)	95.99 ± 3.0	96.54 ± 2.2	0.23

*GIN* gap in noise, *PPI* pre-pulse inhibition, *SS* sodium salicylate, *M* ± *SD* mean ± standard deviation.

Significant P values (P < 0.05) are in bold. Data are represented as M ± SD.

### Electrophysiological tests

**Figure 1:**
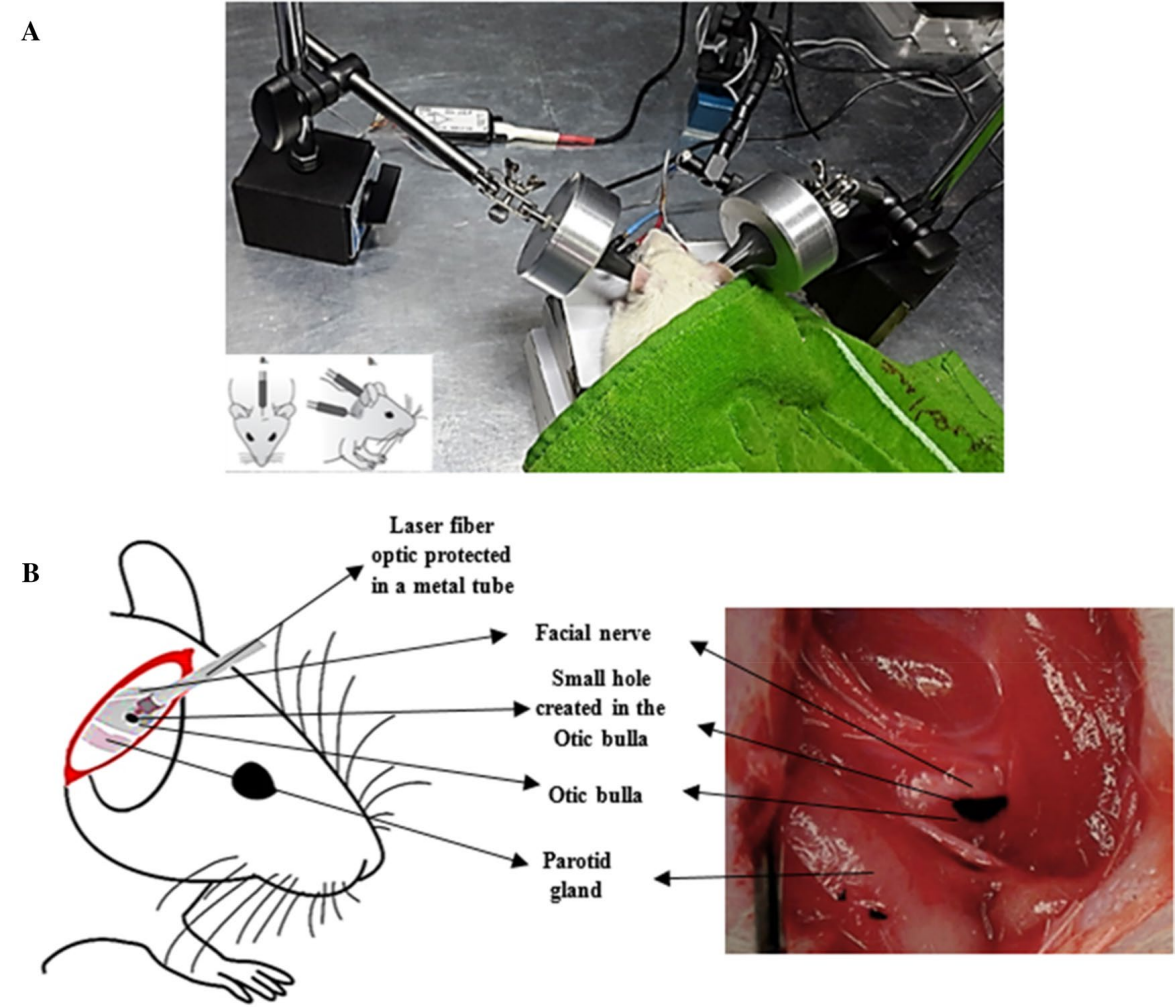
Electrophysiological tests of aABR and oABR using acoustic and optoacoustic stimulations. (**A**) Three surface electrodes were placed on the scalp of the anesthetized animal to record the aABR by the audiology lab device. (**B**) Ear surgery was performed to access the cochlea and laser irradiation for oABR test. The Otic bulla was exposed through a posterior tympanum approach. The facial nerve and parotid gland were the landmarks for finding the Otic bulla, where a small hole was created to expose the cochlea. The Audiology Lab device was synchronized with the laser device to record the evoked potentials immediately after the laser irradiation. The laser fiber optic, which was placed in a metal tube for protection, was fixed by a mechanical holder in a non-contact mode at a distance of 1 mm from the hole created in the Otic bulla in the direction of the cochlea.

The electrophysiological ABR test was performed with acoustic and optoacoustic stimulations. The aABR test was performed for both groups in the first session and also for the tinnitus group after completing the SS injection period (Fig. 1A). After ear surgery to approach the cochlea, oABR test was performed for both groups (Fig. 1B).

### ABR waveforms

**Figure 2:**
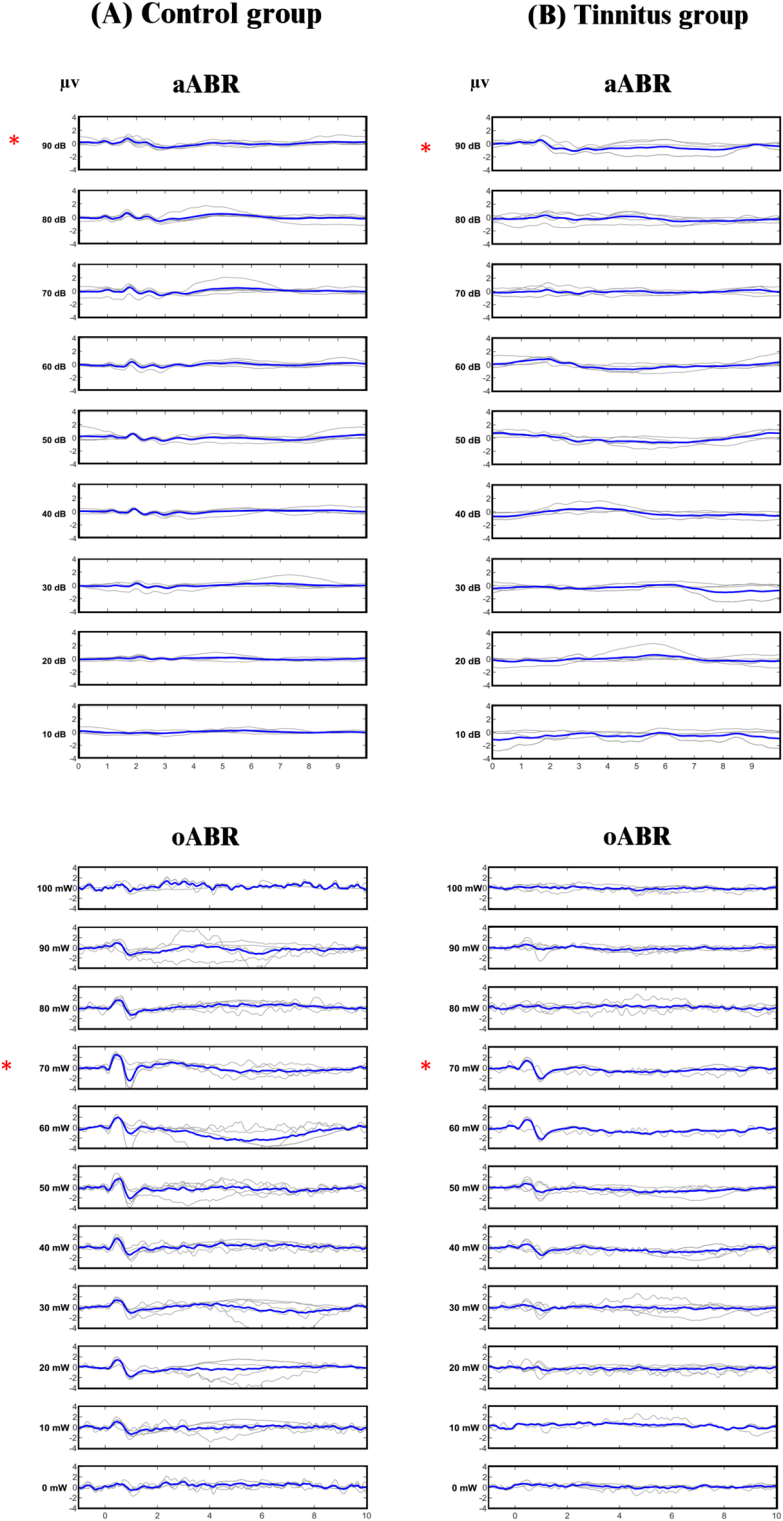
aABR and oABR waveforms. (**A**) Control group, (**B**) tinnitus group. In each row, the bold line represents the grand average of the waveforms. The highest mean amplitude of wave II, which was the most prominent waveform in both aABR and oABR, occurred at 90 dB SPL aABR and 70 mW oABR power, indicated by *.

The first ABR waveforms were generated at 20 decibel sound pressure level (dB SPL) of aABR and 10 milli watt (mW) of oABR (Fig. 2).

### Determination the optimal ABR waveforms

**Figure 3:**
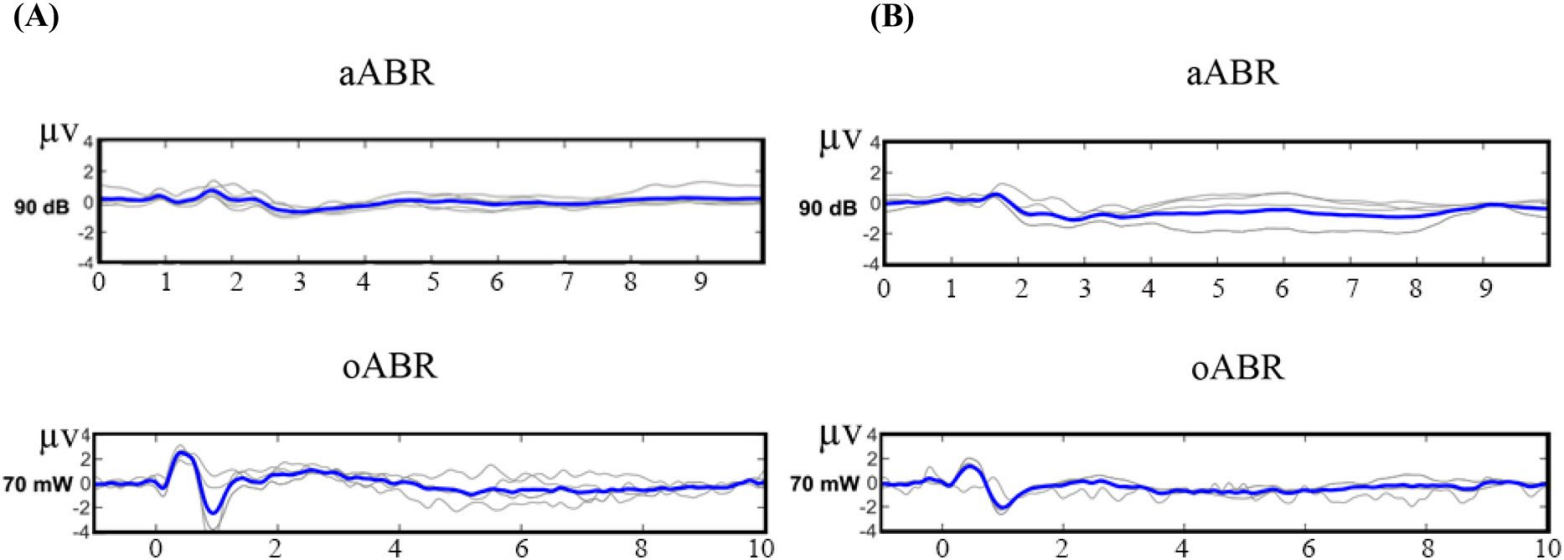
Single waveforms of aABR at 90 dB SPL and oABR at 70 mW from control and tinnitus groups were set side by side for comparison. The bold line in each group represents the grand average. (**A**) Control group, (**B**) Tinnitus group.

In both aABR and oABR, the most prominent wave with the highest mean amplitude was wave II. (Fig. 2) In aABR, the highest mean amplitude of wave II was 0.67 ± 0.03 micro volte (μV) which was produced at 90 dB SPL, and in oABR, the highest mean amplitude of wave II was 2.33 ± 0.5 μV which was produced at 70 mW. Although the amplitude of the waveforms was reduced after tinnitus induction, the highest mean amplitude was still produced at 90 dB SPL aABR and 70 mW oABR (Table 2). Therefore, in this study, the waveforms produced at 90 dB SPL aABR and 70 mW oABR were determined as optimal and used for comparison. To compare the appearance of the waveforms, 90 dB SPL aABR waveforms and 70 mW oABR waveforms of control and tinnitus groups were juxtaposed (Fig. 3).

**Table 2:** Between-groups comparison of aABR and oABR characteristics in control and tinnitus groups (t-test).

Electrophysiological tests									
	aABR		P v.	aABR		P v.	oABR		P v.
	Control	Tinnitus pre-SS		Control	Tinnitus post-SS		Control	Tinnitus	
	M ± SD	M ± SD		M ± SD	M ± SD		M ± SD	M ± SD	
AMP wave I (µV)	0.23 ± 0.18	0.19 ± 0.20	0.84	0.23 ± 0.18	0.21 ± 0.09	0.85	0.39 ± 0.26	0.56 ± 0.29	0.34
AMP wave II	0.67 ± 0.03	0.60 ± 0.11	0.74	0.67 ± 0.03	0.55 ± 0.01	0.13	2.33 ± 0.5	0.71 ± 0.85	**0.003**
AMP wave III	− 0.15 ± 0.16	− 0.18 ± 0.1	0.63	− 0.15 ± 0.16	− 0.03 ± 0.03	**0.005**	0.55 ± 0.28	0.42 ± 0.3	0.5
AMP wave IV	0.36 ± 0.01	0.31 ± 0.06	0.93	0.36 ± 0.01	− 0.21 ± 0.2	0.26	043 ± 0.52	0.28 ± 0.35	0.3
AMP wave V	0.44 ± 0.15	0.34 ± 0.23	1.00	0.44 ± 0.15	0.40 ± 0.08	0.65	0.82 ± 0.65	0.58 ± 0.31	0.8
BTT (ms)	4.37 ± 0.23	4.13 ± 0.08	0.19	4.37 ± 0.23	4.34 ± 0.34	0.95	3.28 ± 0.45	3.56 ± 0.51	0.38
Threshold	16 ± 5.4 (dB)	16 ± 8.2 (dB)	1.00	16 ± 5.4 (dB)	38 ± 7.4 (dB)	**0.001**	16 ± 4.8 (mW)	26 ± 4.4 (mW)	**0.02**

*M* ± *SD* Mean ± standard deviation, *AMP* amplitude, *µV* micro volte, *mW* milli watt, *ms* milliseconds, *SS* sodium salicylate, *dB* decibel, *BTT* brainstem transmission time, *Pv.* P value.

Significant P values (P < 0.05) are in bold.

### Between-groups comparison of ABR characteristics

Between-groups comparison was made between control and tinnitus groups. Mean amplitude of wave III in aABR was − 0.03 ± 0.03 μV in the control group, which was significantly higher than − 0.15 ± 0.16 μV in the tinnitus group (P = 0.005). Mean amplitude of wave II in oABR was 2.33 ± 0.5 µV in the control group which was significantly higher than 0.71 ± 0.8 in the tinnitus group (P = 0.003). Threshold in aABR was 38 ± 7.4 dB SPL in the tinnitus group which was significantly higher than 16 ± 5.4 dB SPL in the control group (P = 0.001). Threshold in oABR was 26 ± 4.4 mW in the tinnitus group which was significantly higher than 16 ± 4.8 mW in the control group (P = 0.01). No significant changes were observed between the control and tinnitus groups in terms of amplitude and brainstem transmission time (BTT) in aABR and oABR tests (Table 2).

### Within-group comparison of ABR characteristics

A within-group comparison was made in the tinnitus group before and after tinnitus induction in terms of aABR characteristics. There were no significant changes in BTT and amplitude, but the mean threshold was significantly increased to 38 ± 7.4 dB after SS injection compared to 16 ± 8.2 dB before injection (P value = 0.0001) (Table 3). A comparison was made within the control group and the tinnitus group separately, in terms of optoacoustic and acoustic stimulations. In the control group the M ± SD of BTT in 90 dB was 4.37 ± 0.04 ms in aABR which was significantly higher than 3.28 ± 0.1 ms in 70 mW of oABR (P = 0.0002). In the tinnitus group, the M ± SD of BTT was 4.35 ± 0.09 ms in aABR which was significantly higher than 3.56 ± 0.2 ms in oABR (P = 0.01). In the control group, the M ± SD of wave II amplitude in oABR was 2.33 ± 0.5 µV which was significantly higher than 0.67 ± 0.03 µV in aABR (P = 0.002). In the tinnitus group, the M ± SD of BTT in aABR was 4.35 ± 0.09 ms which was significantly higher than 3.56 ± 0.2 ms in oABR (P = 0.01) (Table 3).

**Table 3:** Within-group comparison of ABR characteristics (paired t-test).

Electrophysiological test: aABR						
	Within tinnitus group					
	Before SS (mean ± SD)			After SS (mean ± SD)		P v.
AMP of wave I (µV)	0.19 ± 0.20			0.21 ± 0.09		0.66
AMP of wave II	0.60 ± 0.11			0.55 ± 0.01		0.55
AMP of wave III	− 0.18 ± 0.1			− 0.03 ± 0.03		0.99
AMP of wave IV	0.31 ± 0.06			− 0.21 ± 0.2		0.3
AMP of wave V	0.34 ± 0.23			0.40 ± 0.08		0.88
BTT (ms)	4.56 ± 0.13			4.15 ± 0.54		0.62
Threshold (dB)	16 ± 8.2			38 ± 7.4		**0.0001**

Electrophysiological tests: aABR and oABR						
	Within control group (Mean ± SD)			Within tinnitus group (Mean ± SD)		
	Acoustic	Optoacoustic	P v.	Acoustic	Optoacoustic	P v.
BTT (ms)	4.37 ± 0.04	3.28 ± 0.1	**0.0002**	4.35 ± 0.09	3.56 ± 0.2	**0.01**
AMP of wave II (µV)	0.67 ± 0.03	2.33 ± 0.5	**0.002**	0.55 ± 0.00	0.71 ± 0.8	0.34

*AMP* amplitude, *SS* sodium salicylate, *BTT* brainstem transmission time, *ms* milli seconds, *dB* decibel, *µV* microvolts, *Pv.* P value.

Significant P values (P < 0.05) are in bold. Comparisons were made for 90 dB of aABR and 70 mW of oABR, which produced the highest mean waveform amplitudes.

## Discussion

This is the first study that used the optoacoustic stimulation to assess tinnitus. In the last decade, the aABR test has been investigated in many studies as an objective evaluation tool in tinnitus, but more studies are still needed to homogenize the findings^9,10^. In the present study, a group of SS-induced tinnitus rats were investigated through behavioral and electrophysiological tests to identify tinnitus-induced abnormalities and compare the characteristics of aABR and oABR.

The first finding was the occurrence of hidden hearing loss in the tinnitus group. A significant decrease in the GIN ratio after completion of the SS injection period indicated the development of tinnitus, but the PPI ratio remained within normal range, indicating that no clinically detectable hearing loss had occurred (Table 1). Despite normal PPI, mean thresholds in aABR and oABR increased in the tinnitus group, indicating the occurrence of hidden hearing loss (Tables 2, 3). In hidden hearing loss, hearing status is normal in terms of routine hearing tests, but neural output from the cochlea is reduced due to cochlear synaptopathy. This decrease in neural output subsequently returns to normal at the level of the brainstem^35^. It has been reported that the tinnitus-inducing dose of SS can causes cochlear synaptopathy and loss of connections between inner hair cells (IHCs) and afferent auditory nerve fibers^36,37^. On the other hand, it has been indicated that in animal models of hidden hearing loss caused by aging or noise trauma, synapses between IHCs and auditory nerve fibers are permanently lost, but sensitivity to quiet sounds remains unchanged^38^. The pre-pulse in the PPI test, is a short duration noise burst with the same amplitude as the background sound, so a normal PPI ratio despite an increased ABR threshold may be due to remaining the sensitivity to quiet sounds.

Another finding was the significant reduction of the amplitude of wave III in aABR, and the amplitude of wave II in oABR in the tinnitus group compared to the control group (Table 2). Due to the very low amplitude of wave III, which was 0.15 ± 0.16 μV in the control group and 0.03 ± 0.03 μV in the tinnitus group, it is difficult to use the amplitude changes of this waveform as a valid indicator of tinnitus. In our findings, the most prominent wave in both aABR and oABR was wave II, which is similar to the findings of a study performed on Wistar rats^39^. It has been reported that in human subjects in whom tinnitus was associated with normal audiogram, ABR showed a significant reduction of amplitude of wave I produced by primary auditory nerve fibers, but the amplitude of wave V, which is more rostral, remained normal^35^. In animal studies, the most consistent finding in tinnitus subjects with normal hearing was decreased amplitude of wave I^9^. Although the origin of hidden hearing loss is synaptopathy created in the cochlea, there is evidence that the related pathological changes first appear in the cochlear nucleus^40^. The origin of wave I is from the dendrites of the auditory nerve fibers, wave II from the nucleus of the cochlea, wave III from the superior olive complex, and wave IV–V is related to the lateral thalamus. Salicylate has been shown to suppress spontaneous and evoked firing in the dorsal cochlear nucleus. In the present study, the significant reduction of wave II amplitude in aABR after tinnitus induction by SS injection can be explained by the effect of SS on the cochlear nucleus^41^. On the other hand, some pathological changes in the cochlear nucleus can be caused by cochlear synaptopathy due to the hidden hearing loss^42^. Therefore, based on our findings, an increase in threshold in both aABR and oABR and a decrease in amplitude of wave II only in oABR can be the indicators of tinnitus in rats.

The amplitudes recorded in oABR are significantly higher than aABR, one of the reasons for which can be that the intensity in aABR and power in oABR are different, so the 70 dB SPL is not equal to the 70 mW. Another reason may be that the aABR with its lower amplitude may be affected by various conditions such as depth of anesthesia, type of anesthesia, type and age of the animal. Fortunately, the clinically recorded aABR in humans is well known and these problems would not exist in humans. A specific finding to optoacoustic stimulation was the gradual decrease in the amplitude of the waveforms at powers greater than 70 mW, so that it reached the lowest level at 100 mW (Fig. 2). Although IR light has the advantage of a spatially selective effect in stimulating the auditory system, the characteristics of the neural response it evokes may be different from acoustic stimulation. One of the reasons for the gradual decrease in the amplitude of the waveforms can be the accumulation of heat in the tissue at higher powers^15^. Since in human excessive radiation can be harmful or exacerbate tinnitus, it is first necessary to determine what power density is clinically usable in oABR. Considering the potential thermal effects of the optical stimulation, a thermal test should be performed before conducting the oABR test in humans and the minimum power density that can evoke auditory potentials should be determined and used.

The decrease of the amplitude of wave II and increase in threshold after tinnitus induction indicated the important role of hair cells in optoacoustic stimulation. As mentioned, SS at the tinnitus-inducing dose can damage IHCs and the transmission of optoacoustic pressure wave can be disrupt due to the damaged IHCs^36^. Therefore, our results confirm the optoacoustic effect as the main mechanism of optoacoustic stimulation, in which healthy hair cells are required for the pressure wave transmission.

To compare the acoustic and optoacoustic waveform characteristics, the oABR produced more prominent waveforms with shorter BTT. The shorter transmission time may be due to the higher speed of light. In oABR, there was also a significant decrease in the amplitude of wave II due to tinnitus (Table 3). Therefore, in terms of use in the objective diagnosis of tinnitus, oABR has an advantage over aABR due to the production of more prominent waveforms and more changes in tinnitus. Obviously, more animal and human studies are needed to confirm the advantage of oABR over aABR.

A limitation of oABR was the need for ear surgery that requires deep and prolonged anesthesia and could affect the findings, whereas aABR was easily performed by inserting a microphone into the external auditory canal of an anesthetized animal. Due to the small diameter and angularity of the external auditory canal in rats and the 0.8 cm diameter of the laser tube, radiation was hardly possible through the auditory canal and surgery had to be performed to expose the cochlea. Importantly, in human clinical studies, there is no need to surgery and oABR can be performed non-invasively via meatus^29^. Another point is that here, only the tinnitus with hidden hearing loss has been investigated, and tinnitus associated with obvious hearing loss should also be investigated in the future. Also, light-emitting diodes (LEDs) that have a lower price than lasers can be used as the source of optoacoustic stimulation in future studies.

## Conclusion

In this study, acoustic and optoacoustic stimulations have been used in the ABR test for the objective assessment of SS-induced tinnitus. In both aABR and oABR, the highest amplitude was related to wave II. A significant increase in the thresholds of both aABR and oABR and a significant decrease of amplitude of wave II only in oABR were changes that occurred in tinnitus rats. oABR has an advantage over aABR in producing more prominent waveforms, shorter BTT and more changes in tinnitus.

## Materials and methods

### Study design

**Figure 4:**
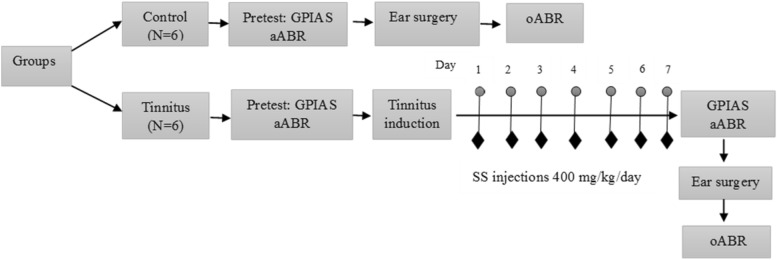
Flow diagram of method and study design. *GPIAS* gap prepulse inhibition of acoustic startle, *GIN* gap-in-noise, *PPI* pre-pulse inhibition, *aABR* acoustic auditory brainstem response, *oABR* optoacoustic auditory brainstem response, *SS* sodium salicylate, *mg* milligram, *kg* kilogram.

First, GPIAS behavioral test and aABR test were performed for the control and tinnitus group. Then, SS was injected in the tinnitus group for 7 consecutive days. The GPIAS and aABR tests were performed again to confirm the occurrence of tinnitus, check the animal’s hearing status and determine the aABR changes. Then ear surgery was performed to access the cochlea and record oABR in both groups (Fig. 4).

### Animals

All procedures of this study were performed in accordance with the National Institutes of Health Guide for the Care and Use of Laboratory Animals and the ARRIVE guidelines. This study was approved by the Experimental Research Committee of the ENT and Head and Neck Research Center of The Five Senses Health Institute of Iran University of Medical Sciences, with the Registration Number of 98-2-22-15523 and also by the Medical Committee of Iran National Science Foundation (INSF) with the code of 91058320. The Ethical Committee of Experimental Sciences of Iran University of Medical Sciences confirmed the study with the ethical code “IR.IUMS.REC.1398.578”. Twelve male Wister rats weighing 180 to 220 g which were born and bred in the Center of Experimental and Comparative Medicine Studies of Iran University of Medical Sciences were divided into control group (n = 6) and tinnitus (n = 6) groups. Rats were raised under standard conditions and housed three rats per cage, with free access to food and water. The ambient temperature was 20 ± 2 °C in a 12-h light/dark cycle. All cages were cleaned at least twice a week. Before the experiment, the cage of the animals was placed in the test room for 30 min to adapt to the surrounding environment.

### Behavioral test

The behavior test was GPIAS that performed by “SR-LAB-Startle test response system”. The GPIAS test measures the GIN ratio, which indicates the occurrence of tinnitus, and the PPI ratio, which assesses the animal’s hearing status. GIN consists of 12 gap trials as well as 12 non-gap trials. Startle reflex occurs when the animal hears an unexpected sound stimulus (pulse) and if the pulse is preceded by a silent gap embedded in the soft background noise, the startle reflex is reduced or actually inhibited. In gap trials, the normal animal detects the gap, which results in a reduction of the startle reflex, but in tinnitus, the tinnitus sound fills the gap and reduction does not occur. GIN ratio is calculated as (pulse − gap/pulse) × 100. Since the GPIAS studies lack common criteria for confirming tinnitus, a precise cut-off value for selecting rats based on percent inhibition of the GIN ratio has not been established and the criterion for proving tinnitus is a statistically significant decrease in GIN ratio compared to before the tinnitus induction^34^. Therefore a significant decrease in GIN ratio indicates the occurrence of tinnitus. The hearing status of the animal should be assessed by the PPI test because if the tinnitus induction method has caused hearing loss, the animal will not be able to detect the gap and thus the GIN test will not be valid. In the PPI test, a weak stimulus (prepulse) is played before the main stimulus (pulse), which is detected by an animal with normal hearing and causes the animal's startle reflex to be inhibited^34,43^.

### Tinnitus induction method

Tinnitus was induced by intraperitoneal injection of 400 mg/kg of sodium salicylate (Sigma-Aldrich, Shanghai, China) diluted in 5 cc/kg of normal saline once a day for 7 consecutive days to the rats in the tinnitus group^30^. Since SS-induced tinnitus is reversible within 72 h, the injection was continued until day 14 of the experiment to maintain the tinnitus pattern. On the 15th day, the GIN values in the tinnitus group showed that the tinnitus was maintained in the animal.

### Electrophysiological tests

Electrophysiological tests included aABR and oABR. Amplitude of five ABR waveforms, BTT and threshold were the parameters measured for both aABR and oABR tests. Amplitude is the distance between peak and trough of each wave, measured in µV. BTT refers to the time between the peak of wave I and the trough of wave V, which represents the progression of nerve stimulation from the distal part of the auditory nerve to the inferior colliculus of the brainstem. The lowest SPL in aABR and the lowest power in mW for oABR at which “consistent” peaks and troughs are observed is the threshold. All the analysis steps were done in a custom-made MATLAB automated program.

### aABR

The ABR was recorded in a soundproof booth in response to the ipsilaterally presented pure tone bursts of 6, 12, and 24 kHz (4-ms duration, 2-ms rise/fall time) at a rate of 21.1 stimuli/s, using an Audiology Lab system (Otoconsult, Frankfurt a. M., Germany) and the recorded waveforms were analyzed offline by MATLAB software to extract the auditory evoked potentials. The ABR waveforms were thus obtained (n = 500 waves) within a time window of 10 ms. The click acoustic stimuli were presented by a calibrated loudspeaker (DT48, Beyer Dynamic, Heilbronn, Germany) via plastic Cone that was placed in the ear meatus of the rat. The surface electrodes were located at the vertex (reference electrode), the mastoid of the left ear (active electrode), and the right ear (ground electrode) (Fig. 1A). As well, electrode impedances ranged from 1 to 3 kΩ for the electrode pairs. The sampling rate was set at 60 kHz and the evoked potentials were filtered from 0.3 to 3.0 kHz. During the measurement, the SPL was decreased from 90 to 10 dB in 10 dB steps to determine the threshold^44,45^.

### oABR

The animals were anesthetized by intraperitoneal injection of Ketamine-Xylazine at dose 100–10 mg/kg. The state of anesthesia was evaluated by pinching the toe every 10 min. Respiration was monitored throughout the procedure to determine when additional supplemental doses were needed. After recording aABR, ear surgery was performed in both groups and the cochlea was successfully exposed through the posterior tympanum approach to record cochlear bioelectric responses. A retroauricular incision was made in the anesthetized animal and after removing the cervico-auricular muscles, the Otic bulla was exposed, in which a small hole of 2 mm in diameter was made. Through this aperture, laser light was irradiated to the cochlea to evoke auditory potentials. The landmarks for finding the Otic bulla were the facial nerve in its superior part and the parotid gland in its inferior part (Fig. 1B).

### Laser characteristics

Laser device “MDL-III-808” from CNI company was used as the optical source. The laser device was coupled to a 200 µm diameter fiber optic shielded in a metal tube. The laser and the “Audiology lab” devices were synchronized to record the auditory evoked potentials immediately after laser irradiation. The wavelength of 808 nm was chosen because of its high penetration depth into the tissue^46^. Vega Ophir laser power meter was used for checking the exact output power after coupling the fiber to the device. The laser parameters, including mode of irradiation, power, pulse width, and repetition rate could be regulated. Laser irradiation was performed in pulse mode with a repetition rate of 10 Hertz (Hz) and a pulse width of 500 microseconds (µs), which were selected according to the optoacoustic articles^16–23^. Power started from 10 mW and increased to 100 mW with 10 mW steps (Fig. 2). The energy range per pulse was from 0 to 50 micro-joules (µJ), increasing in 5 μJ steps (Table 4).

**Table 4:** Parameters of laser.

Wavelength	808 nm
Power (mW)	0 to 100 mW increased with 10 mW step
Power density (mW/cm^2^)	50 to 500 mW/cm^2^
Pulse width (Δt)	500 µs
Repetition rate	10 Hz
Energy/pulse	0 to 50 µJ/P increased with 5 µJ step

### Characteristics of the oABR recordings

NI Signal Express 2015 was used to record the oABR signal at a sampling rate of 50 kHz. The raw data was filtered using a zero-phase Finite Impulse Response (FIR) bandpass filter between 100 and 5000 Hz, and then epoched. The epoched data from the same trials were averaged.

### Statistics

Statistical analysis was done using SPSS software version 24. Descriptive analysis including M ± SD was performed for both behavioral and electrophysiological parameters. Paired t-test was performed for within group comparison and t-test was performed for between groups comparison. P value < 0.05 considered as significant.

## Data Availability

The datasets used and/or analyzed during the current study available from the corresponding author on reasonable request.
